# Adjuvant radiation therapy of regional lymph nodes in breast cancer - a meta-analysis of randomized trials- an update

**DOI:** 10.1186/s13014-015-0568-4

**Published:** 2015-12-21

**Authors:** Wilfried Budach, Edwin Bölke, Kai Kammers, Peter Arne Gerber, Carolin Nestle-Krämling, Christiane Matuschek

**Affiliations:** Department of Radiation Oncology, Medical Faculty, Heinrich Heine University, Dusseldorf, Germany; Department of Biostatistics, Johns Hopkins Bloomberg School of Public Health, Baltimore, USA; Department of Dermatology, Medical Faculty, Heinrich Heine University, Duesseldorf, Germany; Department of Senology, Sana Krankenhaus Gerresheim, Dusseldorf, Germany

**Keywords:** Meta-analysis, Breast cancer, Radiotherapy, Lymph node, Internal mammary, Medial supraclavicular

## Abstract

**Background:**

Adjuvant radiotherapy (RT) of regional lymph nodes (LN) in early breast cancer is still a matter of debate. RT increases the Overall survival (OS) rate of breast cancer patients after breast conserving surgery and after mastectomy in patients with involved LN. The contribution of RT to regional LN to this improvement was poorly identified. Recently, the results of three large randomized trials addressing this question were published as full papers.

**Material and methods:**

Published data of the MA.20 (*n* = 1832), the EORTC22922–10925 (EORTC) (*n* = 4004) trial and the French trial (*n* = 1334) were the foundation of this meta-analysis. Major eligibility criteria were positive i) axillary LN (all trials), ii) LN negative disease with high risk for recurrence (MA.20), and iii) medial/central tumor location (French, EORTC). The MA.20 and the EORTC trial analyzed the effect of additional regional RT to the internal mammary (IM) LN and medial supraclavicular (MS) LN, whereas in the French trial all patients received RT to the MS-LN and solely RT to the IM-LN was randomized. Primary endpoint was OS. Secondary endpoints were disease-free survival (DFS) and distant metastasis free survival (DMFS).

**Results:**

Regional RT of MS-LN and IM-LN (MA.20 and EORTC) resulted in a significant improvement of OS [Hazard Ratio (HR) 0.88 (95 % CL 0.78 - 0.99)]. Adding results of the French trial and using a random effects model to respect the different design of the French trial, the effect on OS of regional RT remained significant [HR 0.90 (95 % CL 0.82 - 0.99)]. The absolute benefits in OS were 1 % in the MA.20 trial at 10 years, 1.6 % in the EORTC trial at 10 years, and 3.3 % in the French trial at 10 years (not significant in single trials). Regional RT of MS-LN and IM-LN (MA.20 and EORTC) yielded to a significant improvement of DFS [HR 0.86 (95 % CL 0.78 - 0.95)] and DMFS [HR 0.84 (95 % CL 0.75 - 0.94)].

**Conclusion:**

Additional regional RT to the internal mammary and medial supraclavicular LN statistically significantly improved DFS, DMFS, and OS in stage I-III breast cancer.

## Introduction

Several clinical investigations showed that breast cancer is a radiosensitive illness [1–6]. There are numerous data which demonstrated that adjuvant RT after breast-conserving surgery minimize the threat of ipsilateral in breast relapse by at least a factor of 3 and halves the risk of any disease recurrence resulting in a significantly improved OS.

But there is still a dispute if regional LN should be radiated in early breast cancer [7–9]. Therefore we performed a meta-analysis to answer this question. In our former meta-analysis we could show that additional regional RT to the internal mammary and medial supraclavicular LN statistically significantly improved DFS, DMFS, and OS in stage I-III breast cancer [7].

Meanwhile results of two randomized studies analyzing these questions have recently been published as full papers [10, 11]. Considering that our previous meta-analysis and thus implications for management of breast cancer therapy was solely based on abstracts of the two studies, we were encouraged to carry out an update on our published results.

## Material and methods

**Fig. 1:**
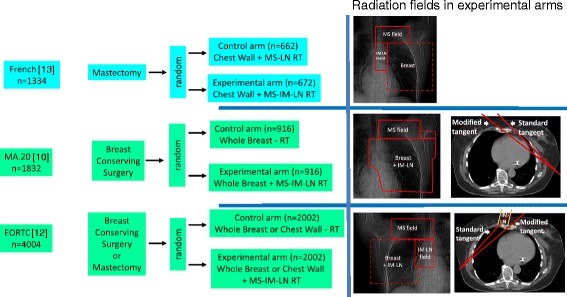
Trial designs. Random = randomization. RT = RT. MS-LN-RT = RT of medial supraclavicular LN. MS-IM-RT = RT of medial supraclavicular and internal mammary LN.

We used the search term “breast cancer” and “RT” and (“regional” or “nodal” or “internal mammary” or “parasternal” or “supraclavicular”) limited to “randomized controlled trial” or “clinical trial, Phase III” in Pubmed (August 2015) yielded 160 publications of possible interest. In addition, we screened abstracts of important annual cancer meetings including meetings of the American Society of Clinical Oncology (ASCO), American Society for Radiation Oncology (ASTRO), European Cancer Congress (ECC), European Society for Radiation Oncology (ESTRO), and San Antonio Breast Cancer Meeting printed between January 2008 and September 2015. The quality of the available information was evaluated according to the Cochrane guidelines. Especially, assigned treatments needed to be done randomly, risk factors between treatment arm evenly distributed, exclusion of patients from the analysis adequate, and analysis performed on an intend to treat basis. Three trials met the criteria to be included. Two papers were published simultaneously in the New England Journal of Medicine in 2015: the MA.20 [10] and the EORTC 22922–10925 trial [12] and one paper from a French group was published in the International Journal of Radiation Oncology Biology, Physics in 2013 [13]. The patient characteristics from these three randomized trials are available (Table 1). Two publications analyzed the impact of additional IM-LN and MS-LN RT and one investigation published the result of additional RT of the IM-LN (Fig. 1). Information regarding the radiation techniques used in the EORTC 22922–10925 and the French trial [13] has been published. In the MA.20 study, MC LN and level 3 axillary LN were treated with an anterior filed. For RT of the IM-LN an adapted wide tangent technique or a direct field corresponding to tangent fields were applied. The inclusion criteria of the studies were similar but not identical (Table 1). Most of the patients had node positive status or medial/central tumors. The primary end point of all trials was the OS rate. Secondary endpoints were DFS, DMFS and loco regional tumor control. Data on DFS and DMFS survival were available solely in the MA.20 and the EORTC 22922–10925, but not in the French investigation [13]. Since data on regional tumor control was presented only in the MA.20, this endpoint was not calculated in the meta-analysis.

**Table 1:** Patients characteristic

	MA.20 [10]	EORTC [12]	French [13]
Recruitment years	2000 - 2007	1996 - 2004	1991 - 1997
Number of patients	1832	4004	1334
Median age	54	54	57
Node positive	90 %	56 %	75 %
Breast surgery	100 % breast conserving	76 % breast conserving	100 % mastectomy
CHX	91 %	55 %	61 %
ER/PR negative	32 %	40 %	7 %
Unknown ER/PR Status	n.a.	n.a.	40 %
Her-2neu	n.a.	n.a.	n.a.
Main inclusion criteria	N+ or high risk* N0 any location	N+ or medial tumor	N+ or medial/central tumor
Breast/chest wall	Both arms:	Both arms:	Both arms: according to practice of the center
	50 Gy/25 fx	50 Gy/25 fx	
Medial supraclavicular nodes	Experimental arm: 45Gy/25 fx	Experimental arm: 50 Gy/25 fx	All patients: dose and fractionation according to practice of the center
Internal mammary nodes	Experimental arm: 45Gy/25 fx	Experimental arm: 50 Gy/25 fx	Experimental arm: 45 Gy/20 fx

*= > = 5 cm tumor, > = 2 cm tumor, and <10 axillary nodes removed with ER-, G3, or lymph vascular invasion; *n.a*. = not available; *fx* = fractions; *ER* = estrogene receptor; *PR* = progesterone receptor

### Statistical analysis

All investigations were stratified by trial. For analysis, hazard ratios with 95 % confidence limits for OS, DFS, and DMFS were extracted from the publications of the MA.20 and the EORTC 22922–10925: Hazard ratio with confidence limits for OS of the French trial was derived from published survival curves according to the method described by Parmar et al. [14], since no information on hazard ratios were given in the publication. Taking into account the detailed information on the patients at risk during follow-up, this method is able to estimate the hazard ratio quite accurately. Meta-analyses of the effect sizes of the MA.20 and the EORTC 22922–10925 trials on OS, DFS and DMFS were carried out using a fixed effect model based on parameter estimates of log hazard ratios in Cox models and their standard errors. Since the project of the French trial was slightly different from the MA.20 and the EORTC 22922–10925 investigations, the combined effect size for OS of all three trials were calculated based on a random effects model. Results are presented with forest plots, in which the estimates of the hazard ratios of all single studies and their combined estimate are visualized. Horizontal bars show the amount of variation (95 % confidence intervals of the parameter estimates). All analyses were performed using MIX 2.0.

## Results

**Fig. 2:**
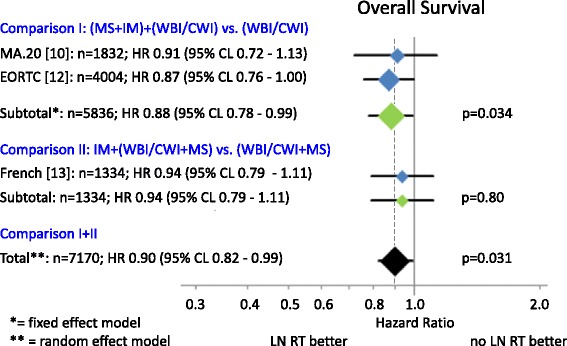
OS. Diamonds are proportional to weights used in meta-analysis, MS + IM = medial supraclavicular and internal mammary lymph node irradiation, WBI/CWI = whole breast irradiation or chest wall irradiation, MS = medial supraclavicular lymph node irradiation.

**Fig. 3:**
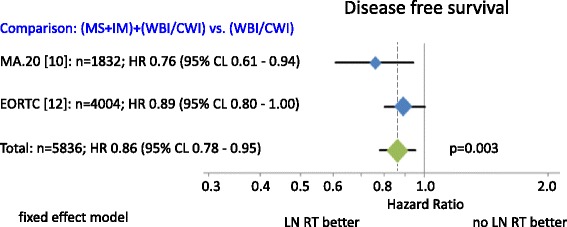
Disease free survivals. Diamonds are proportional to weights used in meta-analysis. MS + IM = medial supraclavicular and internal mammary lymph node irradiation, WBI/CWI = whole breast irradiation or chest wall irradiation.

**Fig. 4:**
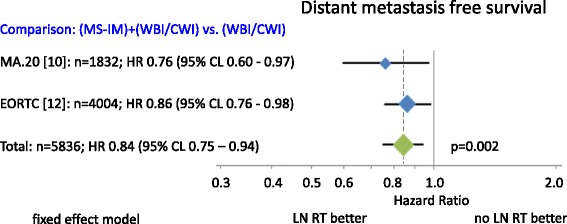
Distant metastases free survival. Diamonds are proportional to weights used in meta-analysis. MS + IM = medial supraclavicular and internal mammary lymph node irradiation, WBI/CWI = whole breast irradiation or chest wall irradiation.

**Fig. 5:**
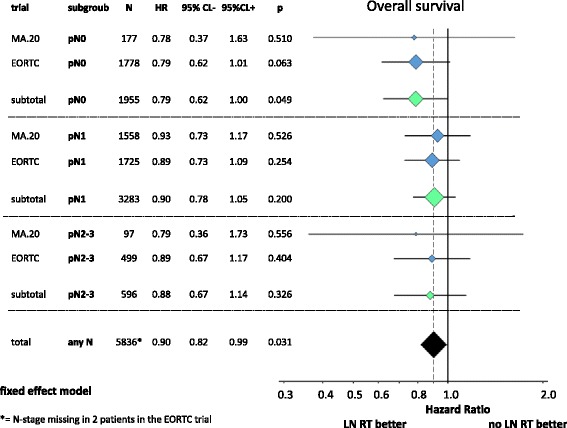
OS for the subgroup analysis. Diamonds are proportional to weights used in meta-analysis

A total of 7170 breast cancer patients from three randomized trials were finally identified for this meta-analysis. Patients’ characteristics in the different trials are presented in Table 1. The regional RT (Fig. 2) of the MS-LN and the IM-LN (MA.20 and EORTC 22922–10925) study lead to a statistically significant improvement of the OS rate in the combined analysis [Hazard Ratio (HR) 0.88 (95 % CL 0.78 - 0.99), *p* = 0.034]. A small, but statistically not significant, improvement in OS was detected in the French trial, in which all patients received MS-RT. In this trial solely the effect of additional RT to IM-LN was tested (HR 0.94 (95 % CL 0.79 - 1.11), *p* = 0.80). Adding the results of the French trial to the results of other trials (Fig. 2) and using a random effects model to take into consideration that the design of the French trial was not identical, the effect on OS of regional RT remained statistically significant (HR 0.90 (95 % CL 0.82 - 0.99), *p* = 0.031). The absolute benefits in OS were 1.6 % in the MA.20 trial at 5 years, 1.6 % in the EORTC 22922–10925 trial at 10 years, and 3.3 % in the French trial 10 years. The results for disease free survival are shown in Fig. 3. LN irradiation leads to a significant improvement when combining the results for the MA.20 and EORTC studies containing *n* = 5836 patients [HR 0.86 (95 % CL 0.78 - 0.95), *p* = 0.003]. In Fig. 4 the results for distant metastasis free survival are shown. Here we could also find a significant improvement for LN irradiation: *n* = 5836; HR 0.84 (95 % CL 0.75 – 0.94, *p* = 0.002). Figure 5 reflects the OS for the subgroup analysis for the MA 20 and the EORTC trial. A slight trend towards a larger improvement in OS from regional RT was observer for patients with no lymph node involvement compared to patients with lymph node involvement. Acute and late side effects are illustrated in Table 2. The absolute numbers for side effects are low in all trials and no grade 4 side effects have been reported.

**Table 2:** Acute and late side effects during radiation therapy

	Ma 20 [10]			EORTC [12]			French [13]		
	MS-IM-	IM-IM+	p	MS-IM-	IM-IM+	p	MS-IM-	IM-IM+	p
Acute side effects									
Fatique grade 2/3	18.2 %	19 %	n.s.	n.a.	n.a.	n.a.	n.a.	n.a.	n.a.
Pain	4.3 %	5.9 %	n.s.	n.a.	n.a.	n.a.	n.a.	n.a.	n.a.
Radiation dermatitis grade 2/3	40.1 %	49.5 %	<0.001	n.a.	n.a.	n.a.	n.a.	n.a.	n.a.
Lung									
Acute side effect (pneumonitis) grade 2	0.2 %	1.2 %	0.01	n.a.	n.a.	n.a.	n.a.	n.a.	n.a.
Acute side effect (pneumonitis) grade 3	0 %	0 %	n.s.	n.a.	n.a.	n.a.	n.a.	n.a.	n.a.
Acute side effect (pneumonitis) grade 4	0 %	0 %	n.s.	n.a.	n.a.	n.a.	n.a.	n.a.	n.a.
Late side effect (fibrosis) grade 2/3	0.3 %	0.4 %	n.s.	n.a.	n.a.	n.a.	n.a.	n.a.	n.a.
Late side effect (fibrosis) grade 4	0 %	0 %	n.s.	n.a.	n.a.	n.a.	n.a.	n.a.	n.a.
Late side effect any grade	n.a.	n.a.	n.a.	1.7 %	4.4 %	<0.0001	n.a.	n.a.	n.a.
Lymphedemia (arm)									
Grade 2/3	4.5 %	8.4 %	0.001	n.a.	n.a.	n.a.	n.a.	n.a.	n.a.
Grade 4	0 %	0 %	n.s.	n.a.	n.a.	n.a.	n.a.	n.a.	n.a.
Any grade	n.a.	n.a.	n.a.	3.6 %	3.8 %	n.s.	n.a.	n.a.	n.a.
Cardiac (any grade)									
Cardiac fibrosis	n.a.	n.a.	n.a.	0.6 %	1.2 %	n.s.	1.7 %	2.2 %	n.s.
Cardiac disease	n.a.	n.a.	n.a.	5.6 %	6.5 %	n.s.	n.a.	n.a.	n.a.
Other cardiac side effects grade 2/3	0.4 %	0.9 %	n.s.	n.a.	n.a.	n.a.	n.a.	n.a.	n.a.
Second cancers (n)	n.a.	n.a.	n.a.	222	191	n.a.	n.a.	n.a.	n.a.
Total late side effects									
Any grade	n.a.	n.a.	n.a.	n.a.	n.a.	n.a.	n.a.	n.a.	n.a.
> grade 2	n.a.	n.a.	n.a.	n.a.	n.a.	n.a.	2.3 %	3.1.%	n.s.

## Discussion

Patient recruitment periods in all studies span back for more than ten years. The combined results of available randomized trials showed a statistically significant improvement in OS for regional nodal RT in stage I-III breast cancer patients (Fig. 2). The total OS benefit at 10 years was comparatively small (1.0 % to 3.3 %). However larger benefits were observed for disease free survival (MA.20: 5.0 %; EORTC: 3.0 %; Fig. 3) and distant metastases free survival (MA.20: 3.9 %; EORTC: 4.0 %; Fig. 4) indicating that a larger advantage may occur with longer follow up. Locoregional tumor control at 10 years was improved by 3.0 % in the MA.20 and by 1.2 % in the EORTC trial. The respective data was not available from the French trial. Possible mechanisms to explain, why the effect of lymph node irradiation (LNI) on disease free survival and distant metastases free survival is larger than on locoregional tumor control were discussed previously [7]. In summary, an under-detection of recurrences in the IMC lymph nodes or immune stimulating effects of LNI are possible explanations.

Unfortunately, with a few exceptions, the fact that different subgroups were analyzed in the 3 trials is making the interpretation of the results and identification of subgroups with larger benefits from LNI difficult. In addition, the results of the few matching subgroups are partially conflicting. According to the MA.20 trial, hormone receptor negative patients have the largest survival advantage from LNI, whereas hormone receptor positive patients had no advantage. Subgroup analysis by hormone receptor status is formally not available from the EORTC trial. However, keeping in mind that almost all patients in the MA.20 trial received chemotherapy, these results are in obvious conflict to observation in the EORTC trial indicating that patients, who had received chemotherapy and endocrine therapy, which should be those with hormone receptor positive tumors and additional high risk features and insofar correspond well to the hormone receptor positive patients in the MA.20 trial, had a large benefit from LNI. To resolve this conflict, joint subgroup analyses of both trials should be initiated and would probably helpful to implement these important results into clinical practice.

Subgroup analyses by lymph node involvement were available from all trials, but sufficient data for a formal meta-analysis available only from the MA.20 and EORTC trial. Patients without involved lymph nodes seem to have a larger advantage from LNI than patient with more than 3 involved lymph nodes (Fig. 5). In the fresh trial, patients with 1–3 involved lymph nodes had a larger survival advantage than pN0 patients. In the MA.20 and the EORTC trial, patients, who underwent a complete axillary dissection (>10 nodes), had consistently a larger benefit from LNI than patients with incomplete axillary dissection. Results according to tumor location (lateral versus central/medial) were inconsistent between trials. Neither data on tumor size nor on tumor grade was available form more than one trial. Overall, there is the impression, but no proof, that patients with higher risk tumors with no or limited lymph node involvement benefit most from LNI.

AntiHer2 therapy was not established at the time, when these trials were initiated.Correspondingly, no information on the Her2-status of the patients is available and trastuzumab was not given to any patient during primary treatment. Accordingly, one can only speculate on the impact of an anti-Her2-therapy on the effect of LNI. Interestingly, a retrospective analysis of the Her2 status of the Danish DBCG 82 b and c trials [15] showed that RT (chest wall + LN) after mastectomy results in no improvement of overall survival in Her2 positive breast cancer, but in a 10 % overall survival gain in hormone receptor positive cancer. This observation indirectly implies that the observed survival benefit from LNI reported here, does not originate from Her2 positive cancer and thus would not be expected to disappear by adding anti-Her2 therapies.

Systemic chemotherapy in the trials on LNI was administered according to the standard treatments at the time of patient’s recruitment (French: 1991–1997. EORTC: 1996–2004, MA.20: 2000–2007). Accordingly, only a few patients in the French trial received anthracyclines and the majority CMF. Although no information is available on the type of chemotherapy in the EORTC trial, one can assume that anthracycline containing regimens were frequently used and in a smaller part of patients taxenes were given. Anthracycline containing chemotherapy was administered in the MA.20 and in approximately in 50 % of those receiving chemotherapy, in addition a taxane was given. Compared to current standards, chemotherapies in the French and in part also in the EORTC trial have to be considered outdated, whereas the chemotherapy in the MA.20 trials could still be regarded as acceptable. The effects of LNI were quite similar between trials indirectly indicating that the effect of LNI may not be substantially changed by more effective chemotherapies. New trials would be necessary for a scientific prove. Since at least 10 year follow up is required in breast cancer to see effects of radiotherapy on overall survival, systemic treatments would likely considered outdated again at the time of publication of such trials. As long as systemic treatments evolve rapidly, physicians have to cope with the fact that indications for radiotherapy are based on trials that did not use the latest standard of systemic treatment. This should not result in an underuse of radiotherapy.

The small benefit of LNI has to be assessed against the additional toxicity. Importantly, no grade IV toxicities were reported. However, LNI is associated with significantly more grade II-III acute skin toxicity and grade I-II lung (Table 2). Grade III lung toxicity was rare (<0.5 %) and not more frequent after LNI. Grade II-III lymphedema was significantly increased after LNI (8.4 % versus 4.5 %) in the MA.20 trials, but not in the EORTC trial. The on average larger radiation fields to the axilla used in the MA.20 trial (Fig. 1) could serve as a plausible explanation for this difference. The fear that radiotherapy to the IMC LN would result in substantially higher cardiac toxicity was not confirmed after already at 10 years follow-up.

One weakness of this meta-analysis is that it is not based on the data of the individual patients, precluding some important subgroup analyses.

In summary, LNI results in a moderate, but statistically significant improvement in disease free survival, distant metastases free survival and overall survival without adding substantial toxicity. Subgroup analyses form published data remain inconclusive, which patients benefit most from LNI.

Based on the available data, it is not easy to give unambiguous recommendations for the implementation of LNI to clinical practice. We think LNI can be advised to patients at higher risk of recurrence, who received systemic chemotherapy in two clinical situations: 1. pN1-pN2 lymph node involvement regardless the tumor location, and 2. pN0 in centrally of medially located tumors. Patients with Her-2 positive tumors, who are treated with trastuzumab and patients with relevant cardiac risk factors should receive supra/infraclavicular lymph node radiation only, but no radiotherapy the internal mammary lymph nodes. Risks and benefits should be discussed in detail.

## Conclusion

Additional regional RT to the internal mammary and medial supraclavicular LN statistically significantly improves DFS, DMFS, and OS in stage I-III breast cancer patients.
